# Choline Alfoscerate in the Treatment of Subthreshold Depression in the Elderly: A Pilot Study (CARTESIO)

**DOI:** 10.3390/jcm15114037

**Published:** 2026-05-22

**Authors:** Filippo Fleishhacker, Annamaria Bonfanti, Nicolò Granata, Claudio Mencacci, Mario Mangrella, Roberto Piazza, Ilaria Coco, Giancarlo Cerveri

**Affiliations:** 1 Dipartimento di Salute Mentale e Dipendenze, ASST Lodi, 26900 Lodi, Italy; annamaria.bonfanti@asst-lodi.it (A.B.); nicolo.granata@asst-lodi.it (N.G.); giancarlo.cerveri@asst-lodi.it (G.C.); 2 Dipartimento di Scienze Mentali e Neuroscienze, ASST Fatebenefratelli, 20157 Milano, Italy; claudio.mencacci@gmail.com; 3 Medical Affairs Department, Italfarmaco S.p.a., 20216 Milan, Italy; m.mangrella@italfarmacogroup.com (M.M.); r.piazza@italfarmacogroup.com (R.P.); i.coco@italfarmacogroup.com (I.C.)

**Keywords:** subthreshold depression, choline alfoscerate, older adults, late-life depression, cholinergic system

## Abstract

**Background**: Subthreshold depression is a prevalent condition among the elderly and often remains untreated due to the limited efficacy and poor tolerability of standard antidepressants. Choline alfoscerate, a cholinergic precursor, is indicated for the treatment of a condition, pseudodepression in the elderly, that is currently clinically classified as subthreshold depression in older adults. Also, choline alfoscerate has shown neuroprotective and antidepressant-like effects. **Objective**: This pilot study aims to evaluate the efficacy and safety of choline alfoscerate in elderly patients with subthreshold depressive symptoms, using contemporary diagnostic criteria and standardized outcome measures. **Methods**: Seventeen patients aged ≥65 years were enrolled in an open-label, single-arm study and received 1200 mg/day of choline alfoscerate for 8 weeks. Clinical and neuropsychological assessments were performed at baseline, after 4 weeks, and at the study’s end. **Results**: A statistically significant improvement was observed in depressive symptoms, as reflected by reductions in HAMD-17 (*p* < 0.001) and GDS-15 scores (*p* < 0.05), as well as in overall clinical severity assessed by the Clinical Global Impression–Severity scale (CGI-S, *p* < 0.05). No significant changes were noted in cognitive performance (MOCA) or apathy (AES-I). The treatment was well tolerated. **Conclusions**: Choline alfoscerate may represent a potentially safe and promising therapeutic option for subthreshold depression in older adults. However, given the exploratory nature of this open-label pilot study, these findings should be considered preliminary and hypothesis-generating and require confirmation in randomized controlled trials.

## 1. Introduction

Subthreshold depression (SD) is a highly prevalent and clinically significant condition among older adults. It is commonly defined as the presence of clinically relevant depressive symptoms that do not meet the full diagnostic criteria for major depressive disorder (MDD) yet are associated with measurable distress and functional impairment [1,2]. In contrast to MDD, SD is characterized by differences in the number, duration, severity, and prevalence of depressive symptoms, which remain below the threshold required for a major depressive episode [3]. Recent meta-analytic evidence indicates that subthreshold depressive symptoms affect nearly 15–20% of older individuals, with higher prevalence rates observed in primary care and institutional settings [4,5]. Importantly, SD is not a benign condition; rather, it represents a clinically meaningful syndrome associated with increased morbidity and reduced quality of life [6].

In late life, subthreshold depressive symptoms have been consistently linked to functional decline, greater healthcare utilization, and increased risk of disability [6,7]. Moreover, longitudinal studies suggest that SD significantly increases the likelihood of progression to major depressive disorder [8]. Beyond affective outcomes, accumulating evidence indicates that late-life depressive symptoms, including those below the diagnostic threshold, are associated with accelerated cognitive decline and heightened risk of mild cognitive impairment and dementia [9,10]. The overlap between affective symptoms, apathy, and executive dysfunction suggests that subthreshold depression may represent an early manifestation of shared neurobiological vulnerability affecting both mood and cognition [11].

Despite its prevalence and clinical consequences, the management of subthreshold depression in older adults remains controversial, and clear clinical guidelines are still lacking [3]. Evidence supporting the use of antidepressants in minor or subthreshold depression is limited, and their efficacy appears modest, particularly in mild symptom presentations [12]. Furthermore, antidepressant treatment in older adults is frequently complicated by adverse effects such as anticholinergic burden, orthostatic hypotension, increased fall risk, fractures, and clinically significant drug–drug interactions in the context of polypharmacy [13,14]. These safety concerns often lead clinicians to adopt a conservative “watchful waiting” approach, leaving many patients undertreated despite persistent symptoms and functional compromise [15].

Given these limitations, there is growing interest in alternative therapeutic strategies characterized by a more favorable safety profile and mechanisms targeting neurobiological pathways implicated in both mood regulation and cognitive function. Choline alfoscerate (L-α-glycerylphosphorylcholine, α-GPC) is a cholinergic drug with well-established efficacy and safety profiles approved for the treatment of so-called pseudodepression in elderly individuals, a terminology historically used in geriatric neuropsychiatry to describe depressive symptomatology occurring in the context of cognitive decline. In contemporary psychiatric and geriatric literature, this construct largely overlaps with what is currently defined as subthreshold depression or minor depression in older adults [3]. α-GPC is capable of increasing central acetylcholine availability by providing bioavailable choline and facilitating its passage across the blood–brain barrier [16], and it has been investigated in cognitive disorders and has demonstrated efficacy in improving cognitive performance in elderly patients with vascular and neurodegenerative conditions [17]. In the ASCOMALVA trial, adjunctive treatment with choline alfoscerate was associated with slower cognitive decline in patients with Alzheimer’s disease receiving donepezil [18].

Beyond its established cognitive effects, emerging preclinical and clinical evidence suggests that choline alfoscerate influences additional neurotransmitter systems relevant to mood regulation. Experimental data indicate that its administration can increase dopamine and serotonin levels in frontal cortical regions [19], suggesting potential modulation of neural circuits involved in motivation, reward processing, and affective regulation. Reductions in apathy and depressive symptoms in patients with Alzheimer’s disease have also been reported in elderly populations treated with choline alfoscerate in combination with donepezil, suggesting potential benefits of enhanced cholinergic stimulation [20,21].

The pathophysiological interplay between depressive symptoms, apathy, and cognitive impairment in older adults supports the hypothesis that interventions enhancing cholinergic transmission while modulating dopaminergic and serotonergic pathways may offer therapeutic benefit [11,22]. However, despite its rationale and indirect clinical evidence, no study to date has investigated the efficacy of choline alfoscerate in elderly patients with subthreshold depressive symptoms. Given the evolution of diagnostic terminology in geriatric psychiatry, it is clinically relevant to investigate whether treatments historically used for “pseudodepressive” states remain effective in patients meeting current criteria for subthreshold late-life depressive symptoms.

The CARTESIO study was therefore designed as an open-label pilot investigation to evaluate the efficacy and safety of choline alfoscerate in older adults with subthreshold depression, using the most recent diagnostic assessments, with particular attention to depressive symptom severity, cognitive performance, and apathy.

## 2. Materials and Methods

### 2.1. Study Design

The CARTESIO study was a monocentric, open-label, single-arm pilot study conducted at the Department of Mental Health, ASST Lodi (Italy). This design was chosen in light of the exploratory nature of the study and the lack of prior clinical data specifically addressing the use of choline alfoscerate in elderly patients with subthreshold depressive symptoms.

The study aimed to evaluate the efficacy and safety of choline alfoscerate in elderly patients with subthreshold depressive symptoms, taking into account contemporary diagnostic and follow-up methodologies, as compared with those available at the time of its authorization for the treatment of pseudodepression in the elderly.

The trial was conducted in accordance with the Declaration of Helsinki and Good Clinical Practice guidelines. The protocol was approved by the competent Ethics Committee and authorized by the Italian Medicines Agency (EUCT Number:2025-520899-25-00). All participants provided written informed consent prior to enrolment.

### 2.2. Participants

Patients aged 65 years or older were consecutively recruited from the Departments of Mental Health and Neurology of ASST Lodi, during routine clinical activity, in order to ensure a representative sample of the target population. Eligible participants were required to present with subthreshold depressive symptoms, defined as the presence of two to four depressive symptoms persisting for at least two consecutive weeks, according to Judd’s criteria [2]. Cognitive integrity sufficient for study participation was ensured by requiring a Mini-Mental State Examination (MMSE) score of 24 or higher. All participants were required to be able to understand the study procedures and provide written informed consent.

Patients were excluded if they met diagnostic criteria for Major Depressive Episode or Dysthymia, or if they had a history of schizophrenia, bipolar disorder, dementia, or other psychotic disorders. Additional exclusion criteria included current or recent substance abuse or dependence, severe organic illness (such as metastatic cancer, stroke, myocardial infarction, or severe neurological disorders), and treatment with antidepressant medication or psychotherapy within two weeks prior to enrolment. Participation in other clinical trials was not permitted. Furthermore, any medical or psychiatric condition considered by the investigator to pose a potential risk to the patient or to interfere with study participation resulted in exclusion.

Seventeen patients meeting the eligibility criteria were enrolled in the study. Two participants discontinued prematurely due to lack of motivation, while 15 completed the 8-week treatment period and were included in the per-protocol analysis.

### 2.3. Intervention

All enrolled participants received choline alfoscerate at a fixed oral dose of 1200 mg/day (600 mg twice daily), administered after breakfast and dinner, for 8 weeks.

Compliance was assessed through pill count at follow-up visits. Acceptable compliance was defined as ≥80% of prescribed doses.

### 2.4. Study Procedures and Assessments

Participants underwent clinical and neuropsychological evaluation at:

- Baseline (T0);
- Week 4 (T1);
- Week 8 (T2, end of treatment).

Outcome assessments were performed by clinicians involved in the study, and no blinding procedures were implemented due to the open-label design.

The primary endpoint was the change in depressive symptom severity from baseline (T0) to week 8 (T2), assessed using the Hamilton Depression Rating Scale—17 items (HAMD-17).

Secondary endpoints included changes from baseline to week 8 in:

- Geriatric Depression Scale—15 items (GDS-15);
- Clinical Global Impression—Severity (CGI-S);
- Montreal Cognitive Assessment (MoCA);
- Apathy Evaluation Scale—Informant (AES-I).

Cognitive screening at baseline also included the MMSE.

Safety and tolerability were evaluated through monitoring of adverse events, physical examination, and vital signs. Adverse events were recorded at each visit and classified according to seriousness and possible relationship with the study treatment.

### 2.5. Statistical Analysis

Given the exploratory nature of the study, no formal sample size calculation was performed.

Continuous variables are presented as mean ± standard deviation (SD). Categorical variables are expressed as frequencies and percentages.

Changes in outcome measures between baseline (T0) and week 8 (T2) were analyzed using paired-sample *t*-tests. Normality of data distribution was formally assessed using the Shapiro–Wilk test, and statistical tests were selected accordingly. In case of non-normal distribution, the Wilcoxon signed-rank test was applied.

The primary efficacy analysis was conducted on the intention-to-treat (ITT) population, defined as all participants who received at least one dose of study medication and had at least one post-baseline assessment. For participants who discontinued prematurely, a last observation carried forward (LOCF) approach was applied.

In addition, a per-protocol (PP) analysis was also performed, including participants who completed the study with adequate compliance (≥80%).

All statistical tests were two-tailed, with a significance level set at *p* < 0.05.

## 3. Results

### 3.1. Participant Characteristics

Seventeen patients meeting the eligibility criteria were enrolled in the study. Two participants discontinued prematurely due to lack of motivation and were included in the intention-to-treat (ITT) analysis using a last observation carried forward (LOCF) approach. Fifteen participants completed the 8-week treatment period and were included in the per-protocol (PP) analysis.

The mean age of the sample was 76.0 ± 7.95 years (range 65–94). The mean duration of education was 10.65 ± 4.53 years. Baseline cognitive screening with the MMSE yielded a mean score of 27.02 ± 1.78, consistent with preserved global cognitive function. However, the mean MoCA score at baseline (22.29 ± 4.65) suggests the possible presence of mild cognitive impairment in a subset of participants, reflecting the greater sensitivity of MoCA in detecting subtle cognitive deficits (Table 1).

At baseline, depressive symptom severity was mild, in accordance with the study design. The mean HAMD-17 score was 8.47 ± 3.13, the mean GDS-15 score was 6.82 ± 2.58, and the mean CGI-S score was 2.76 ± 0.83.

### 3.2. Primary Outcome

The primary efficacy analysis was conducted on the intention-to-treat (ITT) population (n = 17), using a last observation carried forward (LOCF) approach for missing data. A per-protocol (PP) analysis was also performed, including participants who completed the study (n = 15).

**Figure 1:**
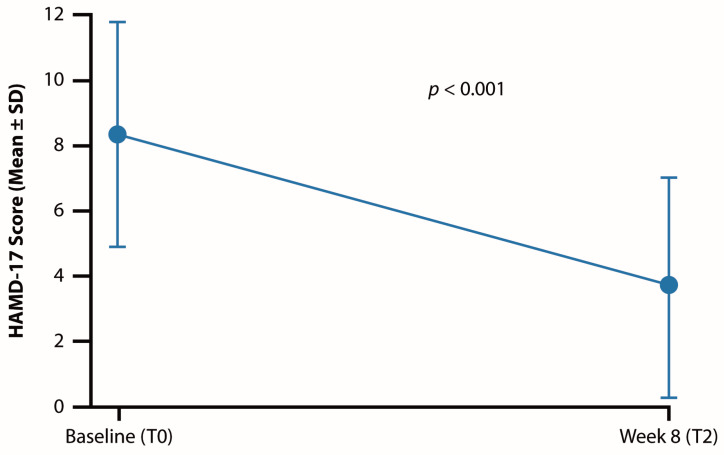
Change in Hamilton Depression Rating Scale (HAMD-17) scores from baseline (T0) to week 8 (T2) in the per-protocol population (n = 15). Values are presented as mean ± standard deviation. A significant reduction in depressive symptom severity was observed after 8 weeks of treatment (paired *t*-test, *p* < 0.001).

In the per-protocol analysis, the mean HAMD-17 score decreased from 8.36 ± 3.43 at baseline to 3.71 ± 3.36 at week 8 (Figure 1), with a statistically significant reduction (paired *t*-test, *p* < 0.001). The magnitude of this effect was large (Cohen’s d = 1.35), indicating a clinically relevant change in depressive symptom severity.

### 3.3. Secondary Outcomes

Changes in secondary efficacy outcomes were also evaluated (see Table 2). The mean GDS-15 score decreased from 6.82 ± 2.58 at baseline to 5.00 ± 3.06 at week 8, indicating a statistically significant reduction in self-reported depressive symptoms (*p* < 0.05). The mean CGI-S score decreased from 2.76 ± 0.83 at baseline to 1.87 ± 0.91 at week 8, indicating a statistically significant improvement in overall clinical severity (*p* < 0.05). Cognitive performance, as measured by the MoCA, showed an increase from 22.29 ± 4.65 at baseline to 25.00 ± 3.78 at week 8 (n = 14); however, this change did not reach statistical significance. Similarly, the mean AES-I score changed from 48.27 ± 5.18 at baseline to 50.07 ± 6.65 at week 8 (n = 15), with no statistically significant difference observed.

### 3.4. Safety and Tolerability

Choline alfoscerate was generally well tolerated. Two adverse events were reported during the study period: one case of headache and one case of transient hypertension. In both instances, symptoms resolved spontaneously. A causal relationship with the study medication could not be definitively established.

No serious adverse events were reported.

## 4. Discussion

The present pilot study investigated the efficacy and safety of choline alfoscerate in elderly patients with subthreshold depressive symptoms. After 8 weeks of treatment, a statistically significant reduction in depressive symptom severity was observed, as measured by the HAMD-17 and GDS-15 scales, together with a significant improvement in overall clinical severity as assessed by the Clinical Global Impression–Severity scale (CGI-S). Treatment was generally well tolerated, with only mild and self-limiting adverse events reported. Given the open-label design and the absence of a control group, these findings should be interpreted as indicative of a signal of efficacy rather than definitive evidence of treatment effectiveness. In this context, it should be noted that placebo response rates in depression studies—particularly in mild or subthreshold conditions—can be substantial, and part of the observed improvement may therefore be attributable to non-specific or placebo-related effects.

### 4.1. Effects on Depressive Symptoms

The significant reduction in depressive symptom severity observed in the present study across multiple clinical measures, including the HAMD-17, GDS-15, and CGI-S scales, suggests that choline alfoscerate may exert a clinically relevant effect on depressive symptoms in late-life subthreshold depression. Even in the presence of mild baseline severity, the observed reduction indicates that subthreshold symptomatology may be responsive to pharmacological modulation targeting non-traditional antidepressant pathways. Importantly, even modest reductions in depressive symptom severity may be clinically meaningful in subthreshold depression, where symptom burden is often associated with functional impairment and reduced quality of life. This interpretation is in line with emerging clinical observations that interventions targeting cholinergic and related neurotransmitter systems may impact on affective symptoms beyond global cognition [3,23].

These findings are particularly relevant in light of the limited and inconsistent evidence supporting the use of conventional antidepressants in minor or subthreshold depression, especially in older adults. In this population, concerns regarding tolerability and adverse effects frequently limit pharmacological intervention [12,13]. The favorable safety profile observed in the present study, therefore, represents an important clinical advantage and is consistent with the relatively benign adverse effect profile of cholinergic precursors reported in elderly populations [3].

From a neurobiological perspective, the observed improvement may be consistent with mechanisms involving cholinergic and monoaminergic modulation. Preclinical and clinical evidence suggest that choline alfoscerate increases dopamine and serotonin availability in frontal cortical regions, which are critically involved in mood regulation, motivation, and executive functioning [3]. Given the overlap between depressive symptoms and early cognitive or motivational impairment in late life, a compound acting on these systems may offer a mechanistically plausible therapeutic effect. Additional recent reviews highlight how α-GPC may improve apathy and affective symptoms through its combined cholinergic and monoaminergic effects, further supporting the biological rationale of our findings [23].

### 4.2. Cognitive Function and Apathy

In contrast to depressive symptoms, no statistically significant changes were observed in cognitive performance (MoCA) or apathy (AES-I) over the 8-week treatment period. Several explanations may account for these findings.

First, the relatively short duration of treatment may have been insufficient to detect measurable cognitive changes, particularly in a population with largely preserved baseline cognitive function. Cognitive improvement in elderly populations, especially when baseline impairment is mild, often requires longer follow-up periods to emerge, as demonstrated in longer-term cholinergic intervention studies [18]. Moreover, depressive symptomatology and cognitive performance do not necessarily evolve in parallel trajectories in late-life conditions [9].

Second, the sample size was limited, consistent with the exploratory nature of the study, thereby reducing statistical power to detect modest effect sizes. Pilot studies are inherently underpowered for small-to-moderate cognitive effects, and findings should therefore be interpreted cautiously [24,25]. The non-significant increase in MoCA scores observed in our sample may represent a signal that warrants investigation in larger, adequately powered randomized controlled trials.

Third, apathy is a multidimensional construct with complex neurobiological underpinnings involving not only cholinergic dysfunction but also frontostriatal dopaminergic circuits [3,20]. While cholinergic modulation may contribute to motivational processes, dopaminergic pathways play a critical role in reward processing and goal-directed behavior. It is therefore plausible that longer exposure, or combination strategies targeting multiple neurotransmitter systems, may be required to observe clinically meaningful changes in apathy. Recent reviews have emphasized the interaction between cholinergic and monoaminergic systems in the regulation of affective and motivational symptoms in aging populations [3].

### 4.3. Clinical Implications

Subthreshold depression in older adults represents a clinically challenging condition. Historically described in part of the geriatric literature as pseudodepressive states, this condition is now more appropriately conceptualized within the spectrum of subthreshold depressive disorders with symptoms not sufficient for a diagnosis of depression. Epidemiological and longitudinal evidence indicate that subthreshold depressive symptoms are associated with an increased risk of progression to major depressive disorder as well as accelerated cognitive decline and incident dementia [4,9,10]. Given these associations, early intervention strategies with favorable tolerability profiles are of particular clinical interest in older populations.

The present findings suggest that choline alfoscerate may represent a potentially safe therapeutic option for elderly individuals with mild depressive symptomatology who may not be ideal candidates for conventional antidepressant therapy [12,13]. While definitive conclusions cannot be drawn from an open-label pilot study with a limited sample size, as methodological literature consistently highlights the nature and limited statistical power of pilot trials [24,25], these findings should therefore be interpreted as preliminary and hypothesis-generating. Recent reviews have also emphasized the therapeutic role of cholinergic precursors such as choline alfoscerate in subthreshold depressive states in older adults, reinforcing the rationale for future controlled studies [3].

### 4.4. Limitations

Several limitations should be acknowledged. Firstly, the study employed an open-label, single-arm design without a control group, which limits causal inference and does not allow exclusion of placebo effects or regression to the mean. In addition, the open-label nature of the study may have introduced expectation bias from both participants and investigators, potentially contributing to the observed improvements in subjective outcome measures. Secondly, the sample size was small, consistent with the exploratory nature of the study, thereby limiting generalizability and statistical power. Thirdly, the relatively short follow-up period may have precluded the detection of delayed cognitive or motivational effects. Finally, although an intention-to-treat approach with LOCF was applied, the handling of missing data may have introduced additional bias, and results should therefore be interpreted with caution.

### 4.5. Future Directions

Future research should include randomized, double-blind, placebo-controlled trials with larger sample sizes and longer follow-up periods to clarify the magnitude and durability of the observed effect of choline alfoscerate in subthreshold depression in elderly patients. It would also be valuable to incorporate functional outcome measures and biomarkers of cholinergic function to better elucidate the mechanistic pathways involved.

## 5. Conclusions

In this open-label pilot study, choline alfoscerate was associated with a significant reduction in depressive symptom severity in elderly patients with subthreshold depression and demonstrated a favorable tolerability profile. However, given the exploratory design and absence of a control group, these findings should be interpreted with caution and considered indicative of a potential signal of efficacy rather than definitive evidence. These findings highlight the role of choline alfoscerate as a therapeutic option for the management of subthreshold depressive states in older populations according to contemporary diagnostic frameworks.

Although no significant effects were observed on cognitive performance or apathy over the 8-week period, the observed signal on depressive symptoms supports further investigation. Larger, randomized, placebo-controlled trials with longer follow-up are warranted to confirm these preliminary findings and to clarify the potential role of choline alfoscerate in preventing progression to major depression or cognitive decline in vulnerable older adults.

## Figures and Tables

**Table 1:** Participants characteristics.

Characteristic	Total Sample (n = 17), Mean ± SD
Age (years)	76.0 ± 7.95
Sex, n (%)	
Male	11 (65%)
Female	6 (35%)
Education (years)	10.65 ± 4.53
MMSE score	27.02 ± 1.78
HAMD-17 score	8.47 ± 3.13
GDS-15 score	6.82 ± 2.58
CGI-S score	2.76 ± 0.83
MoCA score	22.29 ± 4.65

**Table 2:** Secondary Efficacy Outcomes from Baseline (T0) to Week 8 (T2).

Outcome Measure	n	T0, Mean ± SD	T2, Mean ± SD	Mean Change (T2–T0)	*p*-Value
GDS-15	15	6.82 ± 2.58	5.00 ± 3.06	−1.82	<0.05
CGI-S	15	2.76 ± 0.83	1.87 ± 0.91	−0.89	<0.05
MoCA	14	22.29 ± 4.65	25.00 ± 3.78	+2.71	n.s.
AES-I	15	48.27 ± 5.18	50.07 ± 6.65	+1.80	n.s.

Values refer to the per-protocol population (n = 15). GDS-15—Geriatric Depression Scale (15 items); CGI-S—Clinical Global Impression–Severity; MoCA—Montreal Cognitive Assessment; AES-I—Apathy Evaluation Scale–Informant; n.s.—not significant.

## Data Availability

The original contributions presented in the study are included in the article. Further inquiries can be directed to the corresponding author.
